# *Janthinobacterium* CG23_2: Comparative Genome Analysis Reveals Enhanced Environmental Sensing and Transcriptional Regulation for Adaptation to Life in an Antarctic Supraglacial Stream

**DOI:** 10.3390/microorganisms7100454

**Published:** 2019-10-15

**Authors:** Markus Dieser, Heidi J. Smith, Thiruvarangan Ramaraj, Christine M. Foreman

**Affiliations:** 1 Center for Biofilm Engineering, Montana State University, Bozeman, MT 59717, USA; hjsmith12@gmail.com (H.J.S.); cforeman@montana.edu (C.M.F.); 2 Department of Chemical & Biological Engineering, Montana State University, Bozeman, MT 59715, USA; 3 Department of Microbiology & Immunology, Montana State University, Bozeman, MT 59717, USA; 4 School of Computing, College of Computing & Digital Media, DePaul University, Chicago, IL 60604, USA; tramaraj@depaul.edu

**Keywords:** *Janthinobacterium*, comparative genomics, horizontal gene transfer, cold adaptation, environmental sensing

## Abstract

As many bacteria detected in Antarctic environments are neither true psychrophiles nor endemic species, their proliferation in spite of environmental extremes gives rise to genome adaptations. *Janthinobacterium* sp. CG23_2 is a bacterial isolate from the Cotton Glacier stream, Antarctica. To understand how *Janthinobacterium* sp. CG23_2 has adapted to its environment, we investigated its genomic traits in comparison to genomes of 35 published *Janthinobacterium* species. While we hypothesized that genome shrinkage and specialization to narrow ecological niches would be energetically favorable for dwelling in an ephemeral Antarctic stream, the genome of *Janthinobacterium* sp. CG23_2 was on average 1.7 ± 0.6 Mb larger and predicted 1411 ± 499 more coding sequences compared to the other *Janthinobacterium* spp. Putatively identified horizontal gene transfer events contributed 0.92 Mb to the genome size expansion of *Janthinobacterium* sp. CG23_2. Genes with high copy numbers in the species-specific accessory genome of *Janthinobacterium* sp. CG23_2 were associated with environmental sensing, locomotion, response and transcriptional regulation, stress response, and mobile elements—functional categories which also showed molecular adaptation to cold. Our data suggest that genome plasticity and the abundant complementary genes for sensing and responding to the extracellular environment supported the adaptation of *Janthinobacterium* sp. CG23_2 to this extreme environment.

## 1. Introduction

Environments with temperatures permanently below 5 °C dominate the Earth’s biosphere (>80%). While much of the cold biosphere is made up of the world’s oceans, a combined 37% of the land area consists of permafrost regions, 198,000 glaciers, and two ice sheets [1,2,3]. Adaptation to cold temperatures and associated stresses (i.e., desiccation, water activity, radiation, pH, ionic strength, and nutrient availability) [4] allows psychrophilic/psychrotolerant microorganisms to inhabit and even thrive in these extreme and often inhospitable environments. Integrating ‘omics’ technologies with physiological studies on cold adaptation has advanced our understanding of functional and evolutionary biological processes at the molecular level [5,6,7,8]. Specifically, genome comparisons are a powerful tool that enables the exploration of molecular level adaptations in microorganisms inhabiting a broad temperature spectrum. Comparative approaches have identified two overarching trends across bacterial genomes. First, genome size appears to correlate with environmental niches and lifestyle, and second, diversification is explained by horizontal gene transfer [9]. Paradoxically, in genomes from cold-adapted bacterial species both diversification [10,11] and streamlining are observed [6,12], resulting in an increased and decreased genome size, respectively. 

In the present study, we investigated the genomic properties of *Janthinobacterium* sp. CG23_2 [13], a bacterium isolated from a supraglacial stream on the Cotton Glacier, Antarctica. *Janthinobacterium* spp. are frequently isolated from alpine and polar regions [13,14,15,16,17,18,19,20,21,22], while others have been identified as amphibian symbionts [23] or pathogens [24]. The genus *Janthinobacterium* (*Betaproteobacteria*, *Oxalobacteraceae*) includes Gram-negative, motile, aerobic, chemoorganoheterotrophic bacteria that are often resistant to a wide range of antibiotics and heavy metals [16,25,26]. *Janthinobacterium* spp. commonly produce violacein, a purple, water-insoluble secondary metabolite [27]; however, pigment color can vary [14,16,17,19]. 

Unlike most supraglacial streams, which form seasonally in the ablation zone by melt water incisions, the Cotton Glacier stream is a perennial feature. Water only flows during summer melt (4–12 weeks), with variations in stream hydrology related to local daily, seasonal, and interannual climate conditions [28]. The stream bed is devoid of significant amounts of sediments but is flanked by large para-fluvial sediment deposits [29]. Due to the dynamic ephemeral nature of the Cotton Glacier stream, its changing velocity, low in-stream nutrient levels, short residence times, temperatures near or below freezing point, daily freeze–thaw cycles in summer, months of deep-freeze in winter, and extreme transparency to solar UV radiation [28,29], microbial inhabitants must be capable of major metabolic and physiological adjustments year-round. We hypothesized that in order to optimize adaptation to this extremely variable environment, free living microorganisms would form long-lasting metabolic interactions to stabilize their close environment. According to the Black Queen Hypothesis [30], such functional dependencies of sharing genomic resources would select smaller genomes, thereby reducing energetically costly requirements associated with the maintenance of genetic material and metabolic activities; these are genomic features which are characteristic for the oligotrophic environment [31]. Yet, the genome of *Janthinobacterium* sp. CG23_2 was significantly larger than any previously sequenced *Janthinobacterium* genome [13]. To discern the genetic attributes of *Janthinobacterium* sp. CG23_2 that contribute to a larger genome and allow proliferation under these extremely harsh and transient conditions within the Cotton Glacier stream, a comparative genomic approach with 35 *Janthinobacterium* spp. was performed. 

## 2. Materials and Methods

### 2.1. Comparative Genome and Phylogenetic Analyses

The genomes of 35 *Janthinobacterium* species/strains publicly available at the National Center for Biotechnology Information (NCBI) were used for comparison to the *Janthinobacterium* CG23_2 genome (Table 1). Due to the low percentage match to the other *Janthinobacterium* spp. (Table S1), genomes from *Janthinobacterium* spp. B9–8, Marseille, HH01, CG3, and NBRC.102515 were excluded from core gene analysis. Core genes were identified using the algorithm described in Cleary et al. [32], which constructs a compressed de Bruijn graph (CDBG) of a genome population and identifies core genes using the frequently visited regions in the graph. Subsequently, the coding sequences (CDSs) of the *Janthinobacterium* CG23_2 genome were mapped to the core genes using GMAP v1.8 [33]. Default settings were applied except for the “maximum number of paths to show” flag, which was set to 1. The GMAP alignment was filtered for ≥90% query coverage and ≥80% identity. The same parameters were selected in the GMAP v1.8 software package for pairwise comparisons of CDSs identified in the 36 *Janthinobacterium* spp. genomes. 

A *Janthinobacterium* spp. genome tree was inferred using CheckM [34]. CheckM identifies, annotates, concatenates, and aligns 43 marker genes, which were subsequently placed onto a reference genome tree using the implemented pplacer software package [35]. The Maximum Likelihood tree was computed in MEGA7 [36]. The phylogenetic tree was visualized using iTOL version 4.3 [37]. Whole genome sequence in silico DNA–DNA hybridization (DDH) and average nucleotide identity (ANI) between *Janthinobacterium* sp. CG23_2 and *Janthinobacterium* spp. (Table 1) were determined using the default settings for the genome-to-genome distance calculator GGDC 2.1 in combination with the BLAST+ alignment tool [38] and the Average Nucleotide Identity calculator [39,40], respectively. As recommended by the developers [38], results from Formula 2 were considered for DDH, as these estimates are more robust against the use of incomplete draft genomes.

### 2.2. Molecular Analysis of Cold Adaptation

Cold adaptation of proteins from *Janthinobacterium* sp. CG23_2 was identified using a publicly available python script [6]. Two custom databases were generated, using *Janthinobacterium* spp. isolated from mesophilic (31 annotated genomes) and polar/glacial environments (four annotated genomes) (Table 1). The annotated genome from *Janthinobacterium* sp. CG23_2 was compared to these databases using BLASTP [41] with a cutoff score *E*-value ≤10^−15^. Cold-adaptation scores were assigned to each protein based on the following parameters: arginine to lysine ratio; frequency of acidic residues; proline residues; aromaticity; aliphacity; and grand average of hydropathicity (GRAVY) [6]. Cold adaptation was inferred for each index if the direction of change was significant for fewer proline and acidic residues, and lower R/K (arginine/lysine) ratios, aliphacity, aromaticity, and GRAVY. Proteins with ≥3 cold-adaptation indices were determined as being cold-adapted. [6]. Clusters of orthologous groups (COGs) were annotated with WebMGA using default settings [42] and mapped identified COGs against the updated COGs database [43]. 

### 2.3. Horizontal Gene Transfer (HGT)

HGTector [44]; was used to identify putative horizontally transferred genes. All-against-all BLASTP [41] was performed against the NCBI non-redundant protein sequences database with an *E*-value cutoff ≤10^−10^, ≥30% identity, and ≥70% sequence coverage. The protein sequence database, taxonomy database, and a protein-to-taxonomy dictionary were downloaded on August 15, 2018 from the NCBI website. Up to 100 non-redundant hits per protein were preserved. Hits to more than one organism under the same species were excluded. *Janthinobacterium* was defined as the *self* group (NCBI taxonomic ID: 29580), and *Burkholderiales* as the *close* group (NCBI taxonomic ID: 1224). The *distal* group was comprised of all other organisms. Cutoffs of 7.04, 5.55, and 0.79 for the *self*, *close*, and *distal* group, respectively, were computed using the kernel density estimation function. The cutoff in the *self* weight distribution was included to predict putatively HGT-derived genes that were acquired by specific organisms within the *self* group. COGs of predicted HGT-derived genes were annotated with WebMGA using default settings [42] and mapped identified COGs against the updated COGs database [43]. Due to their phylogenetic distance and lack of gene similarity between the other *Janthinobacterium* spp. (0.0–3.8%; Table S1), *Janthinobacterium* spp. Marseille and B9–8 were excluded from this analysis. 

## 3. Results

### 3.1. Genome Statistics of Janthinobacterium spp. 

**Figure 1:**
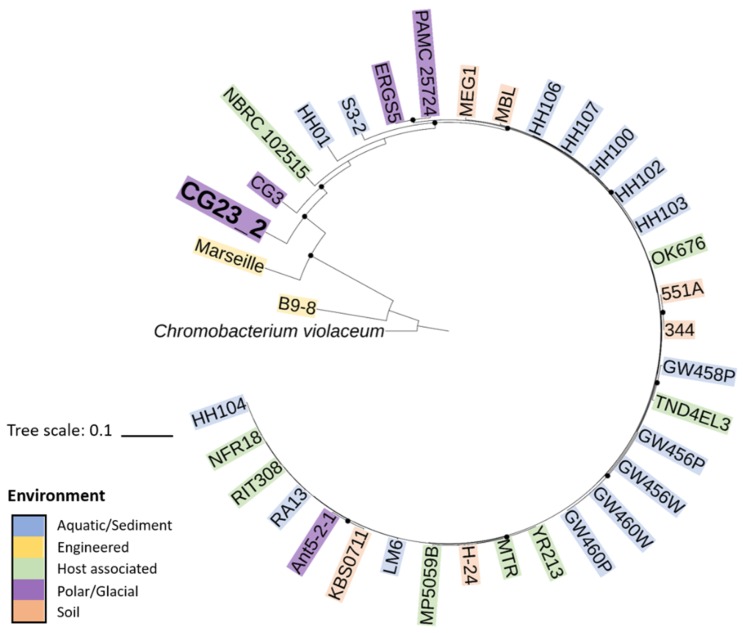
Maximum Likelihood tree of 36 *Janthinobacterium* spp. based on the Whelan And Goldman (WAG) +Freq. model. The tree is rooted to *Chromobacterium violaceum* (i.e., bacterium from closely related genera). The tree with the highest log likelihood (−37179.26) is shown. Circles indicate bootstrap values ≥0.9.

Genome statistics for all 36 *Janthinobacterium* spp. are summarized in Table 1. Genome sizes (4.11–7.85 Mb) and number of CDSs (3870–6859) varied across the analyzed species/strains. All species/strains had a high mole percent GC content (60.5–65.5%), except for *Janthinobacterium* spp. Marseille (54.25) and B9–8 (48.7). *Janthinobacterium* sp. CG23_2 had the largest genome with 7.85 Mb. Its genome was on average 1.7 ± 0.6 Mb larger and predicted 1411 ± 499 more CDSs compared to the other *Janthinobacterium* spp. (Table 1). Calculated DDH and ANI values for *Janthinobacterium* sp. CG23_2 were 21.5 ± 1.8% and 79.3 ± 0.4% (Table S2), respectively, and thus below the threshold boundaries (DDH: 70% and ANI: 95%) for members of the same species. Phylogenetic reconstruction of the 36 *Janthinobacterium* spp. using 43 marker genes identified by CheckM placed *Janthinobacterium* sp. CG23_2 near the root of the tree (Figure 1). *Janthinobacterium* spp. isolated in close proximities (i.e., *Janthinobacterium* spp. CG23_2/CG3; 551a/334; GW spp.; and HH100- spp.) were more closely related. *Janthinobacterium* spp. Marseille and B9–8, the two species with the smallest genome sizes and considerably lower GC contents, were most distantly related to all other members of the genus *Janthinobacterium* investigated (Figure 1 and Figure S1).

**Figure 2:**
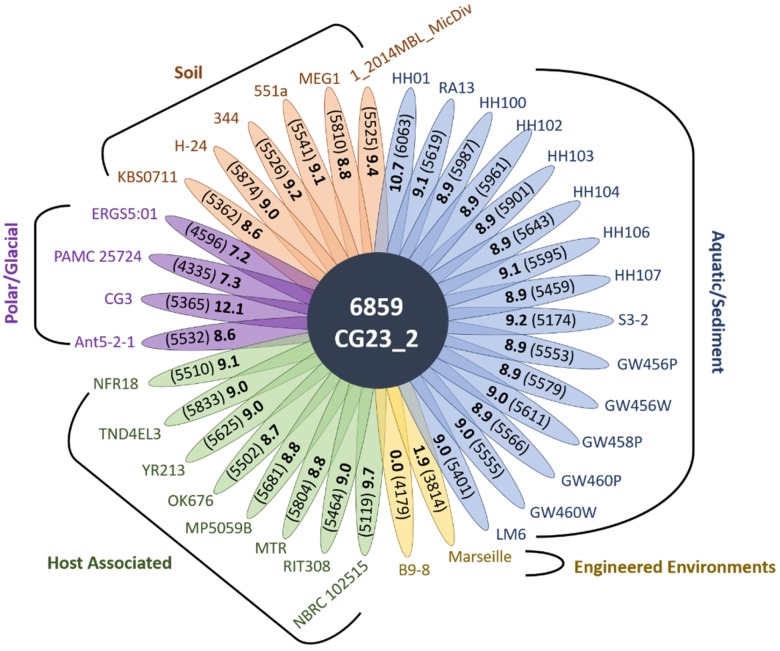
Pairwise gene comparison between *Janthinobacterium* sp. CG23_2 and the 35 *Janthinobacterium* spp. with a 90% query coverage and an 80% identity. The total number of genes from CG23_2 is in the figure center. Each petal depicts the percentage of shared genes (bold) and the total number of predicted protein coding sequences (in parentheses) for each strain.

**Figure 3:**
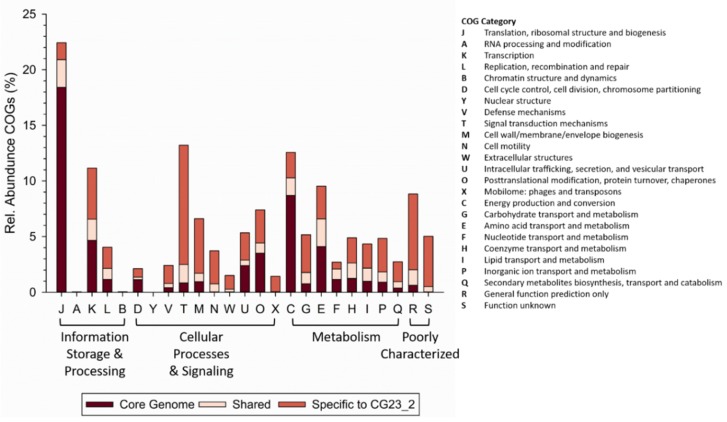
Relative abundance of functional classification of annotated protein coding genes normalized to the total number of protein coding genes for the core genome, shared between *Janthinobacterium* sp. CG23_2 and *n* ≥ 2 species/strains, and species-specific to *Janthinobacterium* sp. CG23_2. Protein coding genes lacking specific functional assignments were excluded. COGs: clusters of orthologous groups.

Collectively, *Janthinobacterium* sp. CG23_2 had only 1282 CDSs (18.7%) in common with the other 35 *Janthinobacterium* spp., as determined by pairwise comparison. Overlap between *Janthinobacterium* sp. CG23_2 and each of the 35 *Janthinobacterium* spp. genomes was low and ranged between 7.2% and 12.1% (Figure 2). Likewise, *Janthinobacterium* spp. B9–8, Marseille, HH01, CG3, and NBRC.102515 had little genome overlap with the other *Janthinobacterium* spp. (0–25% on average; Table S1). These six *Janthinobacterium* spp. with low percentage match (<25%) to the other *Janthinobacterium* spp. were excluded for core gene analysis. For the remaining 30 *Janthinobacterium* spp. 3260 core CDSs were identified. Only 164 CDSs were shared between *Janthinobacterium* sp. CG23_2 and the core genes, indicating the prevalence of the *Janthinobacterium* sp. CG23_2 species-specific accessory genome. Core genes were 12- and 4-fold enriched in the COG categories J and C, respectively, relative to those found specific to *Janthinobacterium* sp. CG23_2 (Figure 3). Conversely, the species-specific accessory genome of *Janthinobacterium* sp. CG23_2 was 13-fold enriched in COG category T, and 4–5 fold in categories V, M, G, and Q, relative to those identified as core genes (Figure 3). COG categories N, W, and X were only identified in the species-specific accessory genome of *Janthinobacterium* sp. CG23_2. 

**Figure 4:**
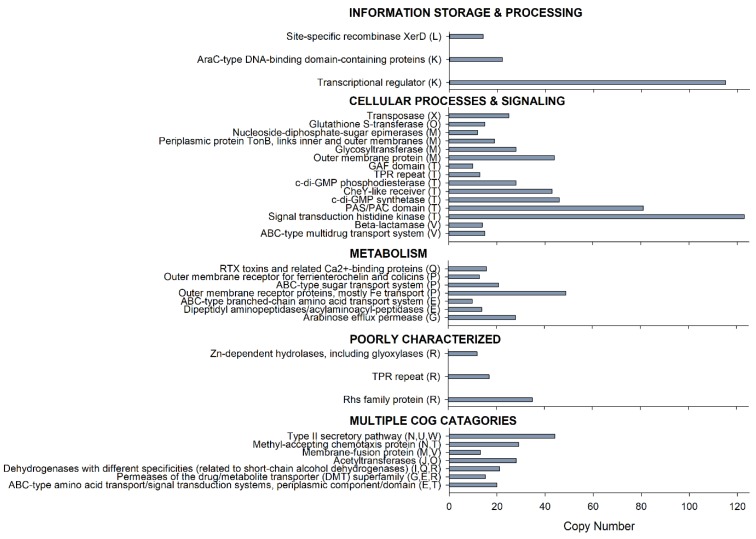
*Janthinobacterium* sp. CG23_2 species-specific clusters of orthologous groups (COGs). Only COGs with *n* ≥10 copy numbers are shown.

The species-specific accessory genome of *Janthinobacterium* sp. CG23_2 contained 81% of the total number of CDS, which equates to 5144 proteins sequences, clustering into 3793 orthologs. COGs with copy numbers *n* ≥ 10 are summarized in Figure 4. The highest percentage of the genes in these COG categories were associated with signal transduction histidine kinases (*n* = 123). Likewise, c-di-GMP synthetases (*n* = 46), c-di-GMP phosphodiesterases (*n* = 28), CheY-like receivers (*n* = 43), and response regulators containing a CheY-like receiver domain (*n* = 84) were prevalent. Further, prominent COGs of the species-specific accessory genome were transcriptional regulators (*n* = 115) and transposases (*n* = 27). Numerous copies of short-chain alcohol dehydrogenases (*n* = 21) and glutathione S-transferases (*n* = 15) were identified. Intercellular competition was mediated by Rhs family proteins (*n* = 35; COG3209). Motility was supported by methyl-accepting chemotaxis protein (*n* = 29), different chemotaxis proteins/regulators/signal transduction proteins (*n* = 19), and a variety of proteins involved in the assembly and function of pili and flagella (*n* = 112). A total of 42 COGs, including lysozymes (*n* = 6), integrases (*n* = 4), terminases (*n* = 3), and genes encoding phage components (*n* = 13) were related to phage proteins.

### 3.2. Genome-Wide Molecular Cold-Adaptation of Janthinobacterium sp. CG23_2

**Figure 5:**
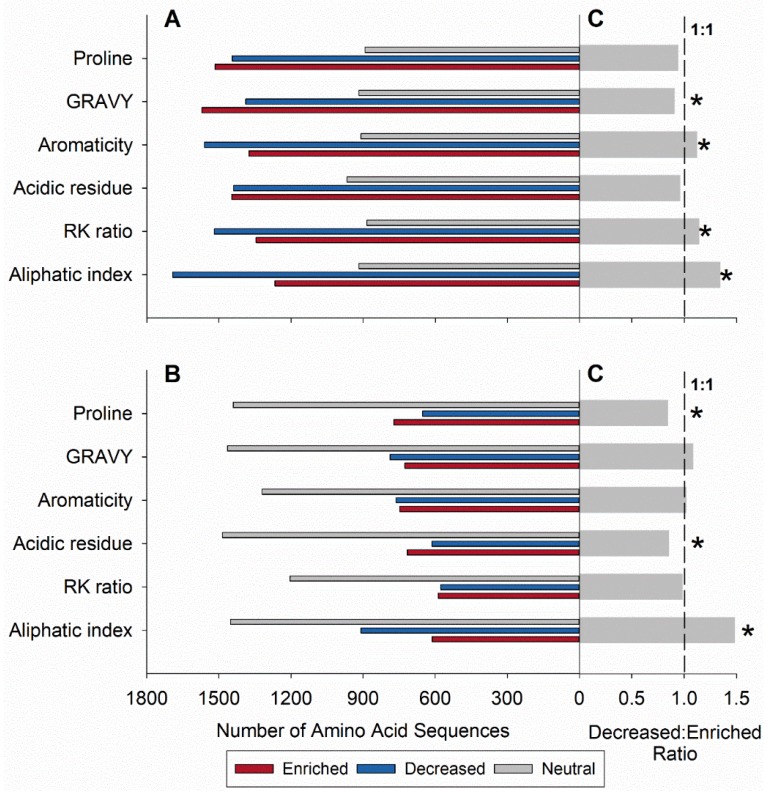
Genome-wide molecular cold-adaptation in CG23_2 compared to (**A**) 31 *Janthinobacterium* spp. isolated from mesophilic environments and (**B**) four *Janthinobacterium* spp. found in polar /glacial regions. (**C**) Adaptation ratios (decreased: enriched) with significant indices are indicated with an asterisk (Bonferroni-corrected *p* ≤ 0.005). Decrease indicates cold adaptation. GRAVY: grand average of hydropathicity; R/K: arginine/lysine.

Cold-adaptation of the entire *Janthinobacterium* sp. CG23_2 genome was inferred from substitution patterns across amino acids using the protein coding sequences of the other 35 *Janthinobacterium* spp. as comparative databases. Across the *Janthinobacterium* sp. CG23_2 genome, 27% (*n* = 1760) and 9% (*n* = 577) of the protein coding sequences indicated cold adaptation when compared to the mesophilic (i.e., 31 *Janthinobacterium* spp.) and polar/glacial (i.e., four *Janthinobacterium* spp.) database, respectively. Noteworthy differences were found in the number of protein coding sequences that were classified as neutral between *Janthinobacterium* sp. CG23_2 and the two databases. The number of protein coding sequences with no significant changes in the amino acid content for *Janthinobacterium* sp. CG23_2 and *Janthinobacterium* spp. isolated from other polar/glacial environments was 2.3 times (*n* = 1687) higher compared to the database built from *Janthinobacterium* spp. isolated from mesophilic environments. Overall, *Janthinobacterium* sp. CG23_2 had significantly more proteins that possessed lower aliphacity, R/K (arginine/lysine) ratios, and aromaticity when compared to their counterparts from mesophilic environments (Bonferroni-corrected *p* ≤ 0.001; Figure 5A–C). Conversely, the GRAVY index was found to be significantly enriched (Bonferroni-corrected *p* = 0.001; Figure 5A–C). When compared to the four *Janthinobacterium* spp. isolated from polar/glacial environments, the *Janthinobacterium* sp. CG23_2 genome was significantly cold-adapted for aliphacity while enriched in proline and acidic residues (Bonferoni corrected *p* ≤ 0.005; Figure 5B,C).

**Figure 6:**
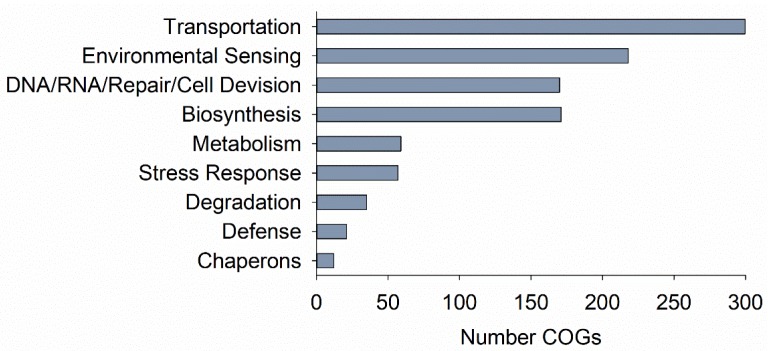
Number of clusters of orthologous groups (COGs) associated with different functions identified for cold-adapted protein sequences in *Janthinobacterium* sp. CG23_2.

COGs were identified for 90% (*n* = 1583) of the cold-adapted protein coding sequences in *Janthinobacterium* sp. CG23_2. Cold-adapted proteins were associated with key processes including transport, environmental sensing, locomotion, defense and stress response, macromolecular syntheses, degradation, and repair, as well as key enzymes in central pathways and their intermediates (Figure 6; Table S3). COGs in high abundance were ABC transporters (i.e., components of the ATP binding cassette) involved in amino acid (*n* = 19; COG0411, COG0683, COG0834, COG1126, COG0765, COG4177, COG0559), multidrug (*n* = 13; COG1131, COG1132), sugar (*n* = 10; COG1129, COG1653, COG3839, COG0395, COG1175), and nitrate/sulfonate/bicarbonate (*n* = 8; COG0715, COG1116, COG0600) transport. Multiple copies of cold-adapted ABC transporters were found for antimicrobial peptides (*n* = 7; COG0577, COG1136) and organic solvent resistance (*n* = 3; COG1127, COG2854, COG0767). Choline dehydrogenases (*n* = 2; COG2303) and the corresponding choline-glycine betaine ABC-type transport system (COG1732) were found to be cold-adapted. Thirty-nine cold-adapted proteins were characterized as outer membrane receptor proteins, mainly involved in iron transport (*n* = 28; COG1629). Cold-adapted efflux pumps such as arabinose efflux permeases (*n* = 17; COG2814), cation/multidrug efflux pumps (*n* = 9; COG0841), and multidrug resistance efflux pumps (*n* = 5; COG1566) were indicative of proteins engaged in transmembrane transport. Secretion of proteins into the extracellular space was represented by 28 copies of Type II secretory proteins (Table S3).

A large number of cold-adapted proteins (*n* = 238) were associated with environmental sensing (Table S3). The genome of *Janthinobacterium* sp. CG23_2 contained multiple copies of CheY chemotaxis proteins (*n* = 5; COG0784) and a methyl-accepting chemotaxis protein (*n* = 16; COG0840). Major proteins involved in sensing of and adaptation to environmental signals were signal transduction histidine kinases (*n* = 48; COG0642) and c-di-GMP synthetases (*n* = 27; COG2199). Three proteins involved in the biosynthesis of histidine were identified as cold-adapted (imidazoleglycerol-phosphate dehydratase (COG0131), histidinol phosphatase (COG0241), and imidazolonepropionase (COG1228)). Cellular response regulators were predominantly CheY-like receivers (*n* = 67; COG3706, COG0745, COG2204, COG3437, COG2197, COG2201). Proteins affecting flagellar activity were associated with the motor (*n* = 3; COG1291, COG1360, COG1536), basal body (*n* = 14; COG2063, COG1766, COG1558, COG1706, COG1261, COG1815, COG1580), and hook (*n* = 9; COG1344, COG1749, COG1256, COG1677) of the flagellar complex. Further, cold adaptation included several proteins involved in flagellar biosynthesis (Table S3) and flagellar biosynthesis chaperones (COG2882, COG1516). Proteins related to environmental stress responses were primarily identified for detoxification (*n* = 17; COG0625, COG0491, COG0346) and protection against oxidative stress (*n* = 20; COG0753, COG1764, COG1225, COG0494, COG0225, COG1858, COG0386, COG0189, COG0695, COG3118, COG0783). Virulence factors such as RTX toxins (*n* = 2; COG2931), Rhs family proteins regulating intercellular competition (*n* = 5; COG3209), Type IV protein secretion systems participating in virulence and antibacterial activities (*n* = 5; COG3519, COG3157, COG3455, COG3523), and beta-lactamases providing antibiotic resistance (*n* = 7; COG1680) were identified as potential defense mechanisms. 

*Janthinobacterium* sp. CG23_2 had 24 and 18 cold-adapted COGs crucial for DNA replication and repair, respectively (Table S3). Overall, 113 proteins denoting 27 COGs were associated with transcriptional functions, dominated by transcriptional regulators (*n* = 72; COG1309, COG1522, COG4977, COG3829, COG1167, COG2909, COG4650). Multiple copies of proteins responsible for methylation (a mechanism protecting newly synthesized DNA from endonucleases) were identified for the methylase of chemotaxis methyl-accepting proteins (*n* = 3; COG1352) and polypeptide chain release factors (*n* = 2; COG2890). 

Several proteins involved in key metabolic steps of the citric acid cycle (TCA) were cold-adapted. These included pyruvate dehydrogenase (COG2609), citrate synthase (COG0372), isocitrate dehydrogenase (COG0473, COG0538), 2-oxoglutarate dehydrogenase (COG0508), and succinate dehydrogenase (COG1053, COG2009). Glucokinase (COG0837), the first step of glycolysis, and multiple copies of short-chain alcohol dehydrogenases (*n* = 18; COG1028, COG4221) and lactate dehydrogenases (*n* = 2; COG1052), both essential during fermentation, were identified. Additionally, proteins supporting carboxydotrophy were cold-adapted (Table S3). Of relevance were proteins efficient in generating precursors or intermediates (e.g., galactose, propionate, glucose, ribulose-5-phosphate, fructose-6-phosphate, pyruvate, acytl-CoA, oxaloacetate) (Table S3) that can be used in catabolic and anabolic pathways to generate ATP or synthesize macromolecular subunits. 

A subset of 48 cold-adapted proteins (Table S3) were involved in the biosynthesis of membrane constituents and included phosphoglycerides (e.g., glycerol-3-phosphate dehydrogenase (*n* = 2; COG0240)), phospholipids (phosphatidylserine synthases (*n* = 4; COG1502), peptidoglycan (e.g., glycosyltransferase (*n* = 6; COG0438), and D-alanine-D-alanine ligase (*n* = 3; COG1181)), fatty acids (e.g., acyl-CoA synthetases (*n* = 4; COG0318) and 3-oxoacyl-(acyl-carrier-protein) synthase (*n* = 3; COG0304)), and lipopolysaccharides (e.g., sugar transferases involved in lipopolysaccharide synthesis (*n* = 3; COG2148)). *Janthinobacterium* sp. CG23_2 had cold-adapted phospholipase (COG3240), amidase (COG3023, COG0860), transglycosylase (COG0741), and peptidase (COG2173, COG1686) required for the continuous remodeling of cellular membranes. Thiol-disulfide isomerase (*n* = 5; COG0526) and acetyltransferases (*n* = 7; COG0456, COG1670), which catalyze protein folding and acetylation, respectively, were among the cold-adapted proteins. Three out of the four proteins involved in beta-oxidation were cold-adapted and included multiple copies of acyl-CoA dehydrogenases (*n* = 8; COG1960), enoyl-CoA hydratase/carnithine racemases (*n* = 3; COG1024), and 3-hydroxyacyl-CoA dehydrogenases (*n* = 3; COG1250). 

### 3.3. Horizontal Gene Transfer in Janthinobacterium sp. CG23_2

**Figure 7:**
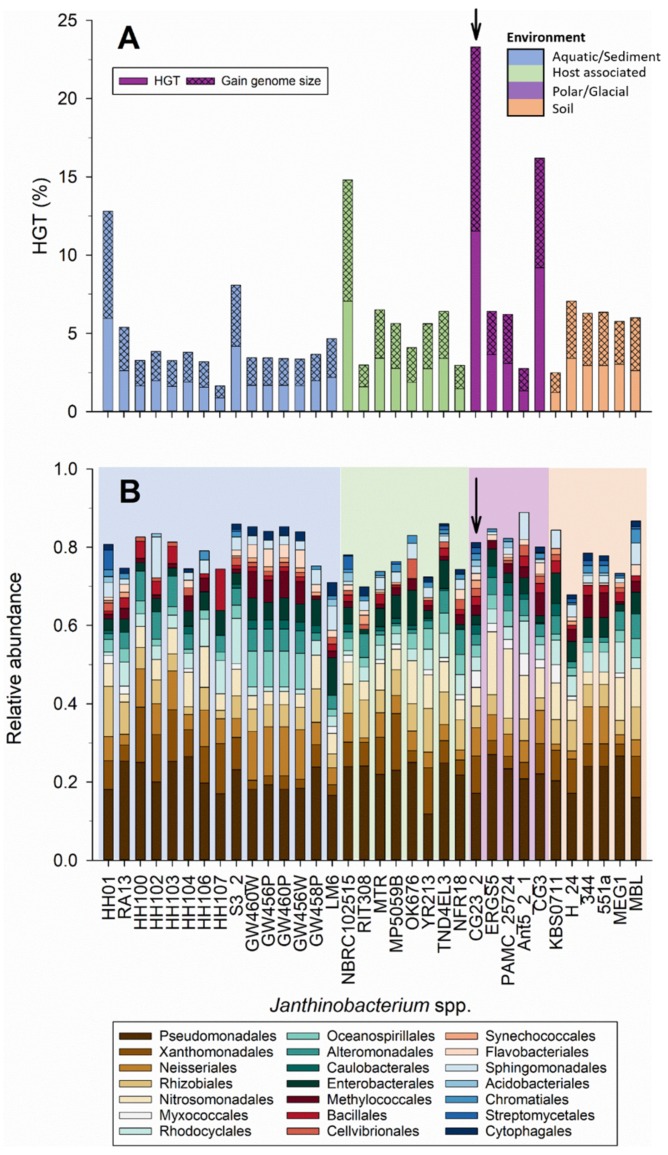
(**A**) Percentage of putatively horizontally transferred genes identified for the 34 *Janthinobacterium* spp. (**B**) Relative abundance of predicted horizontal gene transfer (HGT) donors based on the 20 most abundant orders identified in *Janthinobacterium* sp. CG23_2. Data are presented at the order level. Shaded colors indicate the five environments. Arrows mark *Janthinobacterium* sp. CG23_2.

Putative HGT events across 34 *Janthinobacterium* spp. (excluding Marseille and B9–8) are summarized in Figure 7. *Janthinobacterium* sp. CG23_2 had the highest number of horizontally transferred genes, with 11.5% of its protein coding genes predicted to be the result of HGT events (Figure 7A). Notably, this was 8.8 ± 1.6% above the average for the other *Janthinobacterium* strains. The predicted gain by HGT was 0.92 Mb in *Janthinobacterium* sp. CG23_2, which was substantially higher than the average of 0.17 ± 0.11 Mb for the other *Janthinobacterium* strains (Figure 7A). *Janthinobacterium* spp. that were isolated from the same site such as H100/H103, GW456P,W/GW460P,W, and 551a/344 showed almost identical HGT events (Figure 7B). Genes linked to HGT in *Janthinobacterium* sp. CG23_2 were predominately derived from *Pseudomonadales* (17.1%), *Xanthomonadales* (9.6%), *Neisseriales* (7.3%), *Rhizobiales* (5.5%), and *Nitrosomonadales* (4.8%), similar to the other *Janthinobacterium* species (Figure 7B). Specifically, *Pseudomonas* spp. (*n* = 89), *Rugamonas rubra* (*n* = 28), *Hyalangium minutum* (*n* = 19), and *Lysobacter dokdonensis* DS-58 (*n* = 16) were dominant predicted gene donors. 

Of the 741 putatively identified HGT events in *Janthinobacterium* sp. CG23_2, 292 or 4.5% of the whole genome of *Janthinobacterium* sp. CG23_2 clustered into COGs. COG categories of genes involved in environmental sensing included signal transduction histidine kinases (*n* = 6; COG0642), second messengers (*n* = 5; COG2199), and response regulators (*n* = 3; COG2197) (Table 2). Noticeable were functions associated with defense mechanisms, production of compatible solutes, and the mobilome. These genes comprised Rhs family proteins (*n* = 13; COG3209), RTX toxins and related Ca^2+^-binding proteins (*n* = 7; COG2931), beta-lactamase and other penicillin binding proteins (*n* = 4, COG1680), choline dehydrogenases (*n* = 4; COG2303), phage proteins (*n* = 18), and transposases (*n* = 6) (Table 2). Other abundant HGT-acquired genes included short-chain alcohol dehydrogenases (*n* = 8; COG1028, COG4221) and mannose-6-phosphate isomerases (*n* = 4; COG0662). HTG was predicted for genes encoding for the biosynthesis of peptidoglycan (glycosyltransferase; *n* = 5; COG0438) and fatty acids (3-oxoacyl-(acyl-carrier-protein) synthase; *n* = 4; COG0304). Acetyltransferases (*n* = 6; COG0456, COG1670) with relevance to protein modifications and proteins containing pentapeptide repeats (*n* = 6; COG1357) were of HGT origin. 

## 4. Discussion

Unlike core genomes, which may consist of conserved genes essential to the lifestyle of specific taxonomic groups, the accessory genome is more likely subject to genome evolution and provides selective advantages under specific environmental conditions [45]. Only 18.7% of all CDSs identified in the *Janthinobacterium* sp. CG23_2 genome matched protein sequences to one or more of the other 35 *Janthinobacterium* spp. genomes. Further, with merely 164 CDSs being identified in both the *Janthinobacterium* sp. CG23_2 genome and the core gene set of 30 *Janthinobacterium* spp., these results reinforce the importance of a species-specific accessory genome and the genomic variability of *Janthinobacterium* sp. CG23_2. While it should be noted that gene duplication was not determined, putatively identified HGT events alone increase the genome size of *Janthinobacterium* sp. CG23_2 by 0.92 Mb. HGT events were mainly associated with signal transduction histidine kinases, second messengers, response regulators, and functions linked to defense/stress mechanisms (Table 2). These are all advantageous traits for survival and adaptation to extreme environments (discussed below). HGT is made possible primarily by the mobilome, including transposons and bacteriophages. Notable was the occurrence of 27 COGs denoting transposases in the species-specific accessory genome of *Janthinobacterium* sp. CG23_2, enzymes which catalyze the rearrangement or transfer of mobile genetic elements (i.e., transposons) within or between cells [46]. In a meta-analysis of 384 bacterial genomes, Newton and Bordenstein [47] determined that up to ~6% of bacterial genomes could be the result of bacteriophage genes. These authors also established a correlation between larger genome sizes and an increase in the number of bacteriophage genes. While the *Janthinobacterium* sp. CG23_2 genome is by far the largest genome of the 36 *Janthinobacterium* species investigated, bacteriophage genes account for only 0.6% of its gene content. Smith et al. [48] reported virus to bacterium ratios ranging from 0.12 to 0.44 for the Cotton Glacier stream, 10–1000 fold lower compared to other polar inland waters [49]. Such low viral abundance in the Cotton Glacier stream may have limited the integration of phage genes into the bacterial chromosome of *Janthinobacterium* sp. CG23_2.

The species-specific accessory genome of *Janthinobacterium* sp. CG23_2 is dominated by functions associated with environmental signaling and transcriptional regulation (Figure 4). While both functions are predominant in the core genome of the genus *Janthinobacterium* [22], their enrichment in the species-specific accessory genome of *Janthinobacterium* sp. CG23_2 underscores their role in the adaptation to life in an ephemeral supraglacial Antarctic stream. Moreover, the importance of environmental sensing and orchestrating gene expression were firmly established in the cold-adaptation patterns of protein coding sequences (Table S3). Of relevance were gene categories related to signal transduction histidine kinases and response regulators containing CheY-like receivers. Histidine kinases and response regulators are the building blocks of the two-component signal transduction system, enabling an adaptive response to environmental stimuli (e.g., changes in pH and osmolarity levels, thermal and oxidative stress, light, nutrients and metal ions, and antimicrobials), mainly through gene expression [50]. Moreover, histidine kinases play a central role in the signal integration of the bacterial chemotaxis pathway, where auto-phosphorylated substrates transfer the phosphoryl group to CheY (CheY-P) [51]. Subsequently, the diffusible response regulator CheY-P interacts with the flagellar motor and reverses the rotation of flagella [51]. In line with these findings, the species-specific accessory genome of *Janthinobacterium* sp. CG23_2 possesses chemotaxis genes for sensing environmental cues and the movement towards factors that favor survival. Methyl-accepting chemotaxis proteins were the predominant chemoreceptors: proteins involved in biofilm formation and exopolysaccharide production, flagellum biosynthesis, degradation of xenobiotic compounds, and the production of toxins [52]. In addition to chemotaxis proteins, the presence of c-di-GMP phosphodiesterases and c-di-GMP synthetases suggests the possibility of reciprocal interactions between different chemosensory systems. c-di-GMP, a second messenger, inhibits the methyltransferase activity of methyl-accepting chemotaxis proteins. Ultimately, this modulation affects the phosphorylation of the CheY-like proteins and chemotactic responses [53]. As such, the c-di-GMP signaling system regulates the transition between motile-sessile states [54], lifestyle switches that enhance adaptation to fluctuations in the environment [55]. The species-specific accessory genome of *Janthinobacterium* sp. CG23_2 is equipped with gene categories associated with flagellar biosynthesis, basal body, hook, and motor proteins (*n* = 80) as well as pilus assembly proteins (*n* = 32). While the latter aids the adhesion of a bacterial cell to surfaces, flagella permit chemotaxis-navigated motility systems that allow for active locomotion. Temperature, osmolarity, pH, and nutrient concentration can trigger the expression of the flagellar master operon, which facilitates switching between a motile and sessile state [56]. With their involvement in detecting wetness [57], flagella participate collectively in the sensing of environmental conditions crucial for successful propagation in a supraglacial stream. 

The genome composition of *Janthinobacterium* sp. CG23_2 revealed temperature and oxidative stress as major environmental challenges associated with a supraglacial stream environment. Overall, 1760 and 577 protein coding sequences in the *Janthinobacterium* sp. CG23_2 genome were predicted to be cold-adapted when compared to the *Janthinobacterium* species isolated from mesophilic and polar/glacial habitats, respectively. Both the increased levels of UV radiation above the Antarctic Ice Sheet and low temperatures can lead to the formation of reactive oxygen species, posing a lethal threat to bacterial cells. Protection against oxidative damage in the genome of *Janthinobacterium* sp. CG23_2 included genes such as catalases, hydroperoxide reductases, peroxiredoxins, cytochrome c peroxidases, glutathione peroxidases, glutaredoxins, and thioredoxin reductases, many of which were cold-adapted (Table S3). The species-specific accessory genome of *Janthinobacterium* sp. CG23_2 also contains 15 copies of cold-adapted glutathione S-transferases. Not only do bacterial glutathione transferases provide protection against oxidative stresses, they also play a key role in cellular detoxification including processes such as the biodegradation of xenobiotics and antimicrobial drug resistance [58]. Further, choline dehydrogenases (*n* = 7) were found in the species-specific accessory genome of *Janthinobacterium* sp., and oxidize the first of the two enzymatic steps in the production of glycine-betaine [59]. This compatible solute is a known cryoprotectant and osmolyte and is believed to prevent cold-induced aggregation of proteins and maintain membrane fluidity [59,60]. In addition to genes coping with environmental stresses, Rhs protein families (*n* = 35) are included in the species-specific accessory genome of *Janthinobacterium* sp. CG23_2. Rhs proteins are part of a contact-dependent growth inhibition system. Intercellular competition is mediated by injecting toxins that inhibit the growth of neighboring cells [61], thereby providing a competitive advantage in a low-nutrient environment such as the Cotton Glacier stream [29].

For bacteria to sense and adapt to their ever-changing environment, modifications in signaling and gene regulation pathways are essential [62]. By implication, genotypic selection would depend on the complexity of the environment. Clearly, the temporal heterogeneity of the supraglacial Cotton Glacier stream, Antarctica, poses challenges for its microbial inhabitants within the time frame of both a single and multiple generations. A major survival advantage in this fluctuating environment would be the ability to anticipate changes in the environment [63,64]. Investigations by Mitchell et al. [65], for instance, showed that by using heat shock as the early stimulus, certain bacterial or yeast cells gained protection against stresses to come (e.g., oxidative stress, oxygen depletion). Similarly, a specific response to one stress could increase the resistance to another [66]. While experimental evidence for anticipating stressors or the physiological cross-protection to secondary stresses were beyond the scope of the present study, the genome of *Janthinobacterium* sp. CG23_2 is well equipped with ample genes associated with environmental sensing related functions, transcription regulators, and stress response. 

In the context of the Black Queen Hypothesis, cells can evolve in two ways, by either losing gene functions and mutually depending on other members of a community or by retaining large genomes expressing many genes that are not essential to central metabolism, growth, and reproduction [30]. Although the latter would seem energetically unfavorable in an extreme environment such as the Cotton Glacier stream, *Janthinobacterium* sp. CG23_2 has evolved through genome plasticity (i.e., horizontal gene transfer and transposase activity), features that have been suggested to enable adaptation to life in cold environments [67,68]. The question of whether this gene acquisition represents a common trend in the adaptation to the Cotton Glacier stream environment or *Janthinobacterium* sp. CG23_2 acquired a key status as a function-performing helper within the microbial community according to the Black Queen Hypothesis invites further studies on the network of interactions between co-occurring organisms and their genome evolution. Both whole genome in silico DDH (21.5 ± 1.8%) and ANI (79.3 ± 0.4%) qualified well below the cut-off value for species boundaries [69]. These results were in accordance with the distant branching of *Janthinobacterium* sp. CG23_2 within the Maximum Likelihood tree (Figure 1). Based on these molecular and phylogenetic indicators, *Janthinobacterium* sp. CG23_2 appears sufficiently different to constitute a separate species. The new species *Janthinobacterium cottonii* is proposed. Additional taxonomic studies will help in placing *Janthinobacterium cottonii* within the genus *Janthinobacterium*. 

In conclusion, comparative sequence analysis of 36 *Janthinobacterium* spp. genomes revealed a high degree of speciation of *Janthinobacterium* sp. CG23_2. Initially it was hypothesized that ecological niche specialization would result in genome streamlining; however, the genome of *Janthinobacterium* sp. CG23_2 is significantly larger than other *Janthinobacterium* spp. and has distinct accessory genome features (i.e., environmental sensing, locomotion, response and transcriptional regulation, stress response, and mobile elements) which are well adapted to and suited for proliferation in the ephemeral and extreme conditions of the Antarctic stream. The results highlight how the genome plasticity of closely-related organisms can support the adaptation of individual species to specific environmental niches. 

## Figures and Tables

**Table 1:** Summary of the 36 *Janthinobacterium* genome statistics. (HA: host-associated).

Genome Name	Isolation Source	Size (Mb)	# Contigs	N50 (Mb)	Total # Genes	# Protein Coding Sequences	# 16S rRNA	GC%	NCBI ID (GCA_)
*Janthinobacterium* sp. CG23_2	polar, glacial	7.85	4	4.20	6974	6859	7	63.5	001485665.1
*J.* sp. HH01	aquatic, sediments	7.11	2	4.21	6172	6063	7	64.2	000335815.1
*J.* sp. RA13	aquatic, sediments	6.42	1	6.42	5740	5619	8	62.5	000745325.1
*J.* sp. HH100	aquatic, sediments	6.70	150	0.09	6044	5987	1	62.6	001758685.1
*J*. sp. HH102	aquatic, sediments	6.65	125	0.31	6010	5961	10	62.4	001758625.1
*J*. sp. HH103	aquatic, sediments	6.58	141	0.10	5964	5901	1	62.5	001758705.1
*J*. sp. HH104	aquatic, sediments	6.39	65	0.23	5701	5643	1	62.6	001758715.1
*J*. sp. HH106	aquatic, sediments	6.31	73	0.19	5651	5595	1	62.9	001758725.1
*J*. sp. HH107	aquatic, sediments	6.16	116	0.11	5517	5459	1	63.1	001758765.1
*J. psychrotolerans* sp. S3-2	aquatic, sediments	5.84	62	0.26	5265	5174	8	63.0	001677885.1
*J*. sp. GW456P	aquatic, sediments	6.27	92	0.13	5633	5553	7	62.9	002127655.1
*J*. sp. GW456W	aquatic, sediments	6.26	149	0.08	5660	5579	7	62.9	002127615.1
*J*. sp. GW458P	aquatic, sediments	6.28	157	0.08	6584	5611	4	63.3	002127585.1
*J*. sp. GW460P	aquatic, sediments	6.27	120	0.09	5650	5566	8	62.9	002127625.1
*J*. sp. GW460W	aquatic, sediments	6.27	104	0.11	5637	5555	7	62.9	002127575.1
*J*. sp. LM6	aquatic, sediments	6.29	1	6.29	5523	5401	8	63.0	002002885.1
*J. agaricidamnosum* NBRC 102515	HA: mushroom	5.95	1	5.95	5204	5119	1	61.1	000723165.1
*J. lividum* RIT308	HA: plant	6.21	44	0.30	5554	5464	4	62.8	000632025.1
*J. lividum* MTR	HA: amphibian	6.54	144	0.15	5874	5804	3	62.4	000783415.1
*J.* sp. MP5059B	HA: fungus	6.46	25	0.38	5775	5681	6	62.7	001758645.1
*J.* sp. OK676	HA: plant	6.27	35	0.35	5589	5502	2	62.8	900103595.1
*J.* sp. YR213	HA: root	6.42	16	0.84	5753	5625	6	62.7	900099875.1
*J.* sp. TND4EL3	HA: plant	6.52	78	0.28	5915	5833	6	62.9	900156175.1
*J. lividum* NFR18	HA: root	6.30	26	0.50	5592	5510	2	62.5	900119665.1
*J.* sp. Ant5-2-1	polar, glacial	6.20	1703	0.003	5597	5532	8	62.5	001445815.1
*J.* sp. CG3	polar, glacial	6.27	41	0.78	5460	5365	3	65.5	000344615.1
*J. lividum* PAMC 25724	polar, glacial	4.98	48	0.25	4428	4335	9	60.6	000242815.2
*J. lividum* ERGS5:01	polar, glacial	5.17	16	3.37	4715	4596	8	60.5	001678745.2
*J.* sp. KBS0711	soil	6.07	149	0.09	5438	5362	1	62.7	000988085.1
*J. lividum* H-24	soil	6.71	125	0.12	5916	5874	4	62.4	001758635.1
*J.* sp. 344	soil	6.44	35	0.43	5612	5526	2	63.7	900112025.1
*J.* sp. 551a	soil	6.46	38	0.51	5626	5541	1	63.6	900103675.1
*J. lividum* MEG1	soil	6.60	16	1.11	5893	5810	1	62.3	001854915.1
*J.* sp. 1_2014MBL_MicDiv	soil	6.45	1	6.45	5648	5525	8	63.6	001865675.1
*J*. sp. Marseille	Engineered systems	4.11	1	4.11	3870	3814	2	54.2	000013625.1
*J*. sp. B9-8	Engineered systems	4.73	1	4.73	4295	4179	10	48.7	000969645.2

**Table 2:** Function and clusters of orthologous groups (COG) category for abundant putatively identified HGT events in *Janthinobacterium* sp. CG23_2. (For COG ID description see Table S4).

Function	COG ID and (#) of Protein Coding Sequences
TRANSPORT	
ABC transporters (*n* = 5)	ABC-type amino acid transport/signal transduction systems, periplasmic component/domain: COG0834 (5)
Outer membrane proteins (*n* = 4)	Choline dehydrogenase and related flavoproteins: COG2303 (4)
ENVIRONMENTAL SENSING	
Signaling (*n* = 11)	Signal transduction histidine kinase: COG0642 (6) c-di-GMP synthetase (diguanylate cyclase, GGDEF domain): COG2199 (5)
Response regulator (*n* = 3)	Response regulator containing a CheY-like receiver domain and an HTH DNA-binding domain: COG2197 (3)
DEFENSE (*n* = 27)	RTX toxins and related Ca^2+^-binding proteins: COG2931 (7) Rhs family protein: COG3209 (13) Beta-lactamase class C and other penicillin-binding proteins: COG1680 (4)
MOBILOME (*n* = 24)	Phage proteins: COG3497 (3), COG3772 (2), COG4626 (2), COG4695 (2), COG3500 (1), COG3561 (1), COG3628 (1), COG3645 (1), COG3646 (1), COG3740 (1), COG3948 (1), COG4653 (1), COG5362 (1) Transposase: COG2963 (2), COG3666 (1), COG3335 (1), COG3415 (1), COG2801 (1)
DNA/RNA/REPAIR (*n* = 27)	Superfamily I DNA and RNA helicases: COG0210 (3) Transcriptional regulators: COG1309 (5), COG0583 (5), COG1846 (2), COG1609 (1) AraC-type DNA-binding domain-containing proteins: COG2207 (5)
METABOLISM	
Fermentation (*n* = 8)	Dehydrogenases with different specificities (related to short-chain alcohol dehydrogenases): COG1028 (6) Short-chain alcohol dehydrogenase of unknown specificity: COG4221 (2)
Precursor/intermediates (*n* = 4)	Mannose-6-phosphate isomerase: COG0662 (4)
BIOSYNTHESIS	
Peptidoglycan (*n* = 5) Fatty acids (*n* = 4)	Glycosyltransferase: COG0438 (5) 3-oxoacyl-(acyl-carrier-protein) synthase: COG0304 (4)
Proteins (*n* = 12)	Pentapeptide repeats containing protein: COG1357 (6) Acetyltransferases: COG0456 (3) Acetyltransferases, including *n*-acetylases of ribosomal proteins: COG1670 (3)

## Supplementary Materials

The following are available online at https://www.mdpi.com/2076-2607/7/10/454/s1. Supplemental Figure S1: 16S rRNA gene tree. Supplemental Table S1: Pairwise comparisons of coding sequences (CDSs) identified in 36 *Janthinobacterium* spp. Genomes. Supplemental Table S2: Results for sequences of in silico DNA–DNA hybridization (DDH) and average nucleotide identity (ANI). Supplemental Table S3: Function and COG category for cold-adapted protein-coding sequences in *Janthinobacterium* sp. CG23_2. Supplemental Table S4: List of clusters of orthologous groups (COGs).
