# Supplementary material for: Janthinobacterium CG23_2: Comparative Genome Analysis Reveals Enhanced Environmental Sensing and Transcriptional Regulation for Adaptation to Life in an Antarctic Supraglacial Stream
Source: Microorganisms. 2019 Oct 15;7(10):454. doi: 10.3390/microorganisms7100454 (PMC6843130; doi:10.3390/microorganisms7100454)
Supplement: Supplementary file 1 [file microorganisms-07-00454-s001.zip › Supplemental Table S3.docx]

Table S3: Function and COG category for cold adapted protein-coding sequences in *Janthinobacterium* sp. CG23_2. (For COG ID description see Table S4).

| **Function** | **COG ID and (#) of amino acid sequences** |
| --- | --- |
| **TRANSPORT** |  |
| ABC transporters (n=84) | 1131 (11), 0411 (7), 1136 (4), 0683 (4), 0834 (3), 0577 (3), 1116 (3), 0600 (3), 1653 (3), 3839 (3), 0488 (3), 0715 (2), 1132 (2), 1126 (2), 3842 (2), 1129 (2), 0555 (2), 0765 (1), 2274 (1), 4177 (1), 1120 (1), 0609 (1), 1135 (1), 1464 (1), 4148 (1), 1668 (1), 4555 (1), 1117 (1), 0581 (1), 3639 (1), 4618 (1), 1176 (1), 1177 (1), 0395 (1), 1175 (1), 5265 (1), 1127 (1), 2854 (1), 0767 (1), 1732 (1), 1172 (1), 0559 (1) |
| Efflux pumps (n=31) | 2814 (16), 0841 (9), 1566 (5), 1230 (1) |
| Symporters (n=4) | 2211 (3), 1301 (1) |
| Outer membrane proteins (n=30) | 2885 (9), 1538 (6), 3203 (5), 3713 (3), 3133 (2), 3047 (2), 3248 (1), 3017 (1), 2913 (1) |
| Outer membrane receptors (n=45) | 1629 (28), 4771 (7), 0810 (6), 4774 (3), 4773 (1) |
| Secretion (n=27) | 2165 (7), 1459 (6), 2804 (4), 3267 (3), 4796 (3), 1450 (2), 4795 (1), 1989 (1) |
| Membrane proteins (n=21) | 0845 (8), 5009 (2), 1477 (2), 4942 (2), 0744 (1), 4953 (1), 0481 (1), 4260 (1), 0330 (1), 1585 (1), 2244 (1) |
| Electron transfer (n=29) | 0604 (5), 0277 (3), 2863 (2), 2146 (2), 2010 (1), 2193 (2), 1271 (1), 1294 (1), 4654 (1), 3175 (1), 2124 (1), 0437 (1), 0633 (1), 0056 (1), 0055 (1), 0355 (1), 0356 (1), 0711 (1), 0655 (1) |
| Other transporters (n=20) | 0697 (5), 0475 (3), 0531 (2), 2217 (2), 0848 (2), 0811 (2), 1638 (1), 3090 (1), 0004 (1), 0735 (1) |
| **ENVIRONMENTAL SENSING** | |
| Chemotaxis/chemosensory (n=26) | 0840 (16), 0784 (5), 0643 (3), 1776 (1), 3143 (1) |
| Signaling (n=89) | 0642 (48), 2199 (27), 4191 (4), 2202 (4), 1956 (1), 5000 (1), 0835 (1), 3852 (1), 2206 (1), 5581 (1) |
| Response regulator (n=72) | 0745 (21), 3706 (19), 2204 (12), 2197 (7), 3437 (5), 2201 (3), 3279 (3), 3707 (2) |
| Locomotion-flagellar (n=26) | 1344 (6), 2063 (3), 1766 (3), 1558 (3), 1706 (2), 1261 (1), 1815 (1), 1580 (1), 1749 (1), 1256 (1), 1677 (1), 1291 (1), 1360 (1), 1536 (1) |
| Pilus (n=25) | 5008 (3), 3745 (2), 4965 (2), 2064 (2), 4964 (2), 3063 (2), 4972 (2), 4961 (1), 4970 (1), 4963 (1), 3170 (1), 4968 (1), 3167 (1), 3168 (1), 4726 (1), 2805 (1), 3419 (1) |
| **DEFENSE** (n=21) | 3209 (5), 1680 (7), 2931 (2), 0515 (2), 3519 (2), 3157 (1), 3455 (1), 3523 (1) |
| **STRESS RESPONSE** (n=57) | 0625 (7), 0664 (5), 0491 (5), 0346 (5), 0457 (3), 0753 (3), 1764 (3), 1225 (3), 0790 (3), 0494 (3), 0589 (2), 0225 (2), 1278 (1), 1825 (1), 1188 (1), 3109 (1), 1858 (1), 3210 (1), 0386 (1), 0189 (1), 0695 (1), 3118 (1), 0492 (1), 0631 (1), 0783 (1) |
| **DNA/RNA/REPAIR/CELL DEVISION** | |
| Replication (n=24) | 0513 (4), 4974 (3), 0305 (2), 0188 (2), 0514 (2), 0749 (1), 0587 (1), 0847 (1), 0358 (1), 4973 (1), 1961 (1), 0187 (1), 0553 (1), 0593 (1), 0272 (1), 0632 (1) |
| Repair (n=18) | 0467 (2), 0708 (2), 0420 (1), 1381 (1), 0210 (1), 1112 (1), 1197 (1), 0692 (1), 1573 (1), 0389 (1), 0249 (1), 3663 (1), 1864 (1), 0178 (1), 2818 (1), 0122 (1) |
| Transcription/translation (n=110) | 1309 (40), 1522 (21), 2207 (7), 4977 (6), 0568 (3), 1943 (3), 2916 (3), 1191 (2), 1595 (2), 3829 (2), 1167 (2), 0480 (2), 0060 (2), 0008 (2), 1741 (2), 2909 (1), 3327 (1), 3901 (1), 0215 (1), 0042 (1), 4650 (1), 0154 (1), 0324 (1), 0430 (1), 0242 (1), 0776 (1) |
| Post-transcription (n=9) | 0564 (2), 1187 (2), 0130 (1), 0030 (1), 0144 (1), 0566 (1), 2501 (1) |
| Degradation (n=10) | 0084 (2), 1530 (2), 0507 (1), 1875 (1), 1295 (1), 0349 (1), 0328 (1), 0164 (1) |
| Methylation (n=7) | 1352 (3), 2890 (2), 0350 (1), 0863 (1) |
| Cell division (n=13) | 3116 (4), 0489 (3), 0424 (2), 1475 (2), 0772 (1), 2891 (1) |
| **CHAPERONS** (n=12) | 0542 (3), 0443 (3), 0265 (1), 0071 (1), 0576 (1), 1281 (1), 2882 (1), 1516 (1) |
| **METABOLISM** |  |
| TCA cycle (n=9) | 0508 (2), 0372 (1), 0473 (1), 0538 (1), 1249 (1), 1053 (1), 2009 (1), 2609 (1) |
| Calvin cycle/PPP (n=1) | 0120 (1) |
| Glycolysis (n=1) | 0837 (1) |
| Fermentation (n=23) | 1028 (15), 4221 (3), 1052 (2), 1064 (2), 1062 (1) |
| Aerobic carboxidotrophy (n=5) | 2080 (2), 1529 (2), 1319 (1) |
| Precursor/intermediates (n=33) | 0451 (5), 3386 (3), 3338 (3), 0183 (3), 0663 (2), 2133 (2), 1171 (2), 0662 (1), 0686 (1), 0221 (1), 0836 (1), 0800 (1), 1607 (1), 1472 (1), 1087 (1), 0449 (1), 3265 (1), 2065 (1), 0176 (1), 2301 (1) |

Table S3: Continued

| **Function** | **COG ID and (#) of amino acid sequences** |
| --- | --- |
| **BIOSYNTHESIS** |  |
| Cell wall  Phosphoglycerides (n=8)  Phospholipids (n=8)  Peptidoglycan (n=13)  Fatty acids (n=15)  Lipopolysaccharides (n=4)  Cell wall turnover (n=8) | 0584 (2), 0240 (2), 0204 (2), 0818 (1), 0554 (1)  1502 (4), 3540 (2), 0575 (1), 0688 (1)  0438 (6), 1181 (3), 0812 (1), 0796 (1), 0773 (1), 2348 (1)  0318 (4), 0304 (3), 0331 (2), 0365 (1), 0236 (1), 1043 (1), 2030 (1), 4281 (1), 0427 (1)  2148 (3), 1109 (1)  0741 (2), 1686 (2), 3240 (1), 3023 (1), 0860 (1), 2173 (1) |
| Proteins  Proteins (n=6)  Protein folding (n=7)  Post-translational modification (n=10) | 1020 (1), 1186 (1), 0682 (1), 1952 (1), 0690 (1), 1862 (1)  0526 (5), 1651 (1), 4232 (1)  0456 (4), 1670 (3), 1182 (2), 1247 (1) |
| Amino acids (n=20) | 0367 (2), 0031 (2), 1246 (2), 1045 (2), 0241 (2), 1228 (2), 0065 (1), 2008 (1), 1794 (1), 0436 (1), 0119 (1), 0135 (1), 2309 (1), 0131 (1) |
| Nucleosides (n=7) | 0213 (1), 0125 (1), 0207 (1), 0104 (1), 0299 (1), 0107 (1), 0134 (1) |
| Flagellar (n=7) | 1298 (2), 1684 (2), 1377 (1), 1987 (1), 1157 (1) |
| Ribosomes (n=6) | 1358 (1), 0244 (1), 0256 (1), 0335 (1), 0052 (1), 0049 (1) |
| Vitamins (n=7) | 0161 (3), 0352 (1), 0028 (1), 0054 (1), 0414 (1) |
| Glutamate and glutamine (n=7) | 0493 (2), 0067 (1), 0347 (1), 0174 (1) |
| Molybdenum cofactor (n=8) | 2896 (3), 0303 (1), 0521 (1), 0314 (1), 1763 (1), 0746 (1) |
| Ubiquinone (n=10) | 0654 (4), 2226 (4), 2227 (2) |
| **DEGRADATION** |  |
| Beta oxidation (n=14) | 1960 (8), 1024 (3), 1250 (3) |
| Aldehydes (n=6) | 1012 (6) |
| Proteins (n=9) | 1506 (5), 0616 (2), 0265 (2) |
| Glycine (n=4) | 0665 (4) |
| Organic acids (n=3) | 0657 (3) |
| **NUTRIENTS** |  |
| Sulphur (n=7) | 0607 (4), 2041 (1), 0369 (1), 4117 (1) |
| Nitrogen (n=3) | 1140 (1), 2180 (1), 3256 (1) |
