# Supplementary figures and images for: Janthinobacterium CG23_2: Comparative Genome Analysis Reveals Enhanced Environmental Sensing and Transcriptional Regulation for Adaptation to Life in an Antarctic Supraglacial Stream
Source: Microorganisms. 2019 Oct 15;7(10):454. doi: 10.3390/microorganisms7100454 (PMC6843130; doi:10.3390/microorganisms7100454)

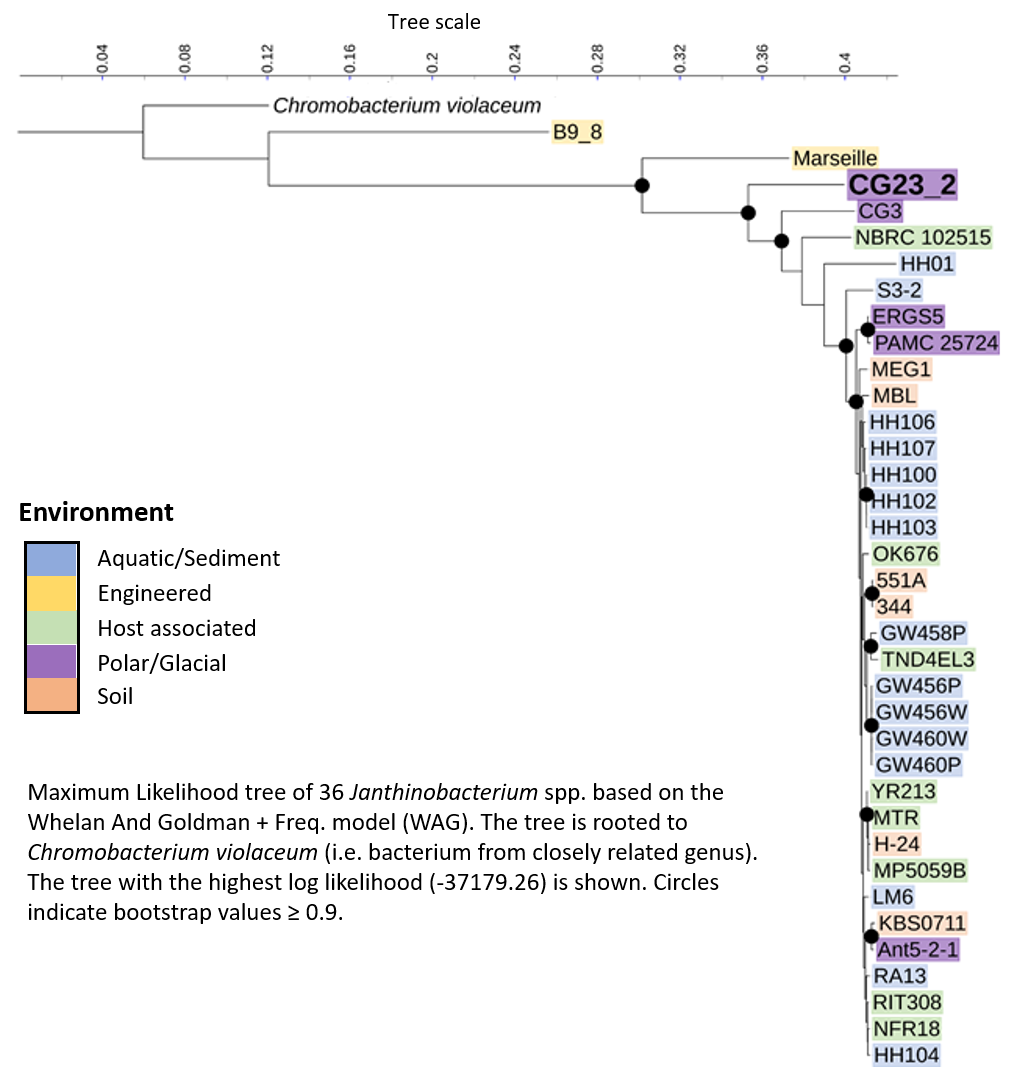

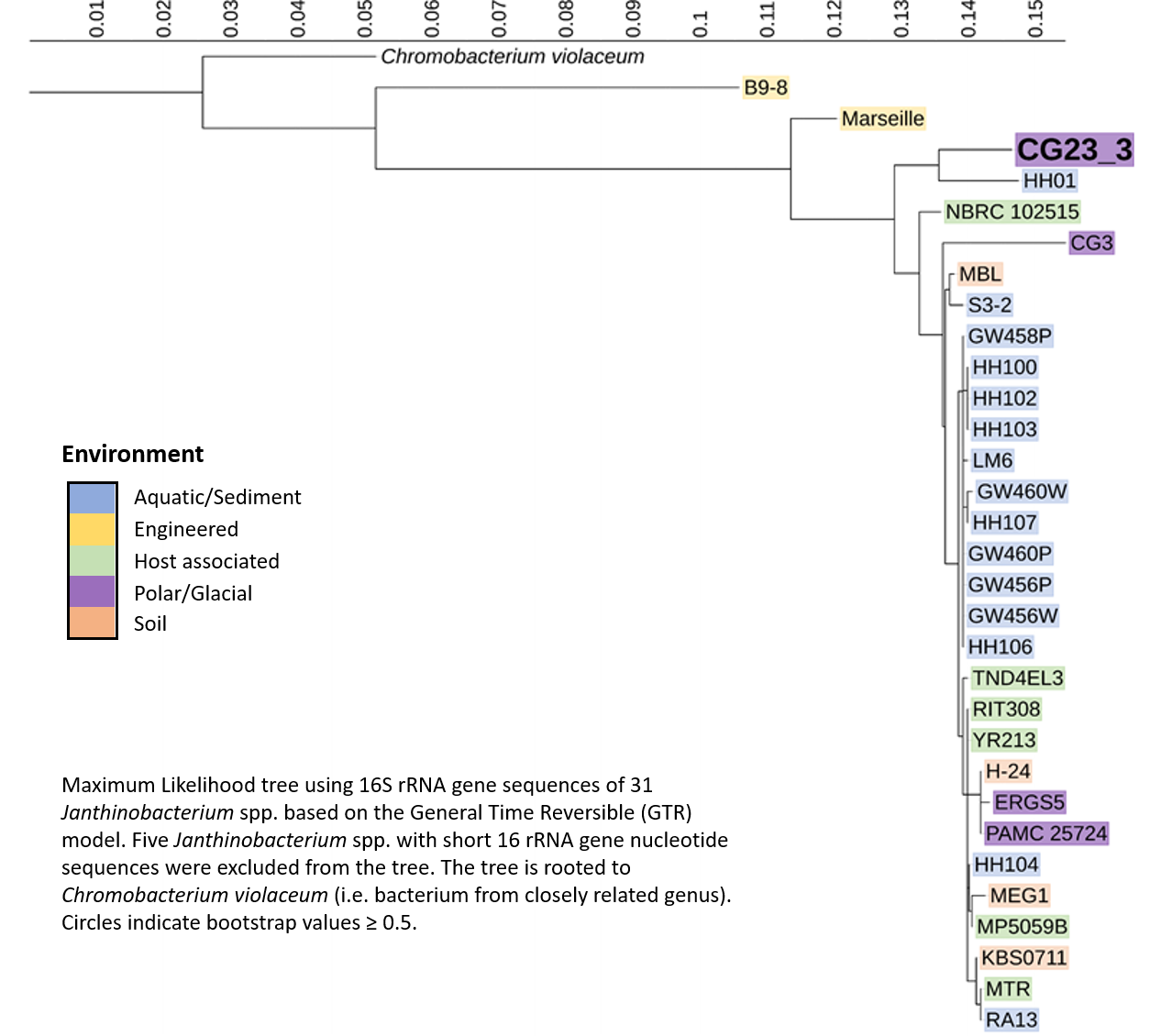

Supplement: Supplementary file 1 [file microorganisms-07-00454-s001.zip › Proof_Supplemental_Figure S1.docx]
